# Footprint area analysis of binary imaged *Cupriavidus necator* cells to study PHB production at balanced, transient, and limited growth conditions in a cascade process

**DOI:** 10.1007/s00253-016-7844-6

**Published:** 2016-10-03

**Authors:** Denis Vadlja, Martin Koller, Mario Novak, Gerhart Braunegg, Predrag Horvat

**Affiliations:** 1 Department of Biochemical Engineering, Faculty of Food Technology and Biotechnology, University of Zagreb, Pierottijeva 6, 10000 Zagreb, Croatia; 2 Office of Research Management and Service, c/o Institute of Chemistry, NAWI Graz, University of Graz, Heinrichstrasse 28/III, 8010 Graz, Austria; 3 ARENA Arbeitsgemeinschaft für Ressourcenschonende & Nachhaltige Technologien, Inffeldgasse 21b, 8010 Graz, Austria

**Keywords:** Binary imaging, Bioreactor cascade, *Cupriavidus necator*, Footprint area analysis, PHA granules, Poly[3-(*R*)-hydroxybutyrate] (PHB)

## Abstract

Statistical distribution of cell and poly[3-(*R*)-hydroxybutyrate] (PHB) granule size and number of granules per cell are investigated for PHB production in a five-stage cascade (5CSTR). Electron microscopic pictures of cells from individual cascade stages (R1–R5) were converted to binary pictures to visualize footprint areas for polyhydroxyalkanoate (PHA) and non-PHA biomass. Results for each stage were correlated to the corresponding experimentally determined kinetics (specific growth rate *μ* and specific productivity *π*). Log-normal distribution describes PHA granule size dissimilarity, whereas for R1 and R4, gamma distribution best reflects the situation. R1, devoted to balanced biomass synthesis, predominately contains cells with rather small granules, whereas with increasing residence time *τ*, maximum and average granule sizes by trend increase, approaching an upper limit determined by the cell’s geometry. Generally, an increase of intracellular PHA content and ratio of granule to cell area slow down along the cascade. Further, the number of granules per cell decreases with increasing *τ*. Data for *μ* and *π* obtained by binary picture analysis correlate well with the experimental results. The work describes long-term continuous PHA production under balanced, transient, and nutrient-deficient conditions, as well as their reflection on the granules size, granule number, and cell structure on the microscopic level.

**Electronic supplementary material:**

The online version of this article (doi:10.1007/s00253-016-7844-6) contains supplementary material, which is available to authorized users.

## Introduction

Polyhydroxyalkanoates (PHA) are a structurally versatile group of polyoxoesters accumulated by various prokaryotes as intracellular storage granules. The homopolyester poly[3-(*R*)-hydroxybutyrate] (PHB), object of this study, constitutes the best investigated PHA. Their production, biogenesis, biodegradation, physicochemical properties, fields of application, and biological functions are exhaustively described (Chen 2009; Jendrossek and Handrick 2002; Koller et al. 2010; Steinbüchel and Valentin 1995; Tan et al. 2014; Zinn et al. 2001). In this context, biogenesis and physiological tasks of PHA granules are of increasing relevance. Only recently, denominating PHA granules as “carbonosomes” was suggested by Jendrossek and Pfeiffer (2014) to reflect their high complexity. Due to their interactive proteinaceous surface compounds (Grage et al. 2009; Mayer and Hoppert 1997), they are much more than just simple, inert, polymeric spheres, as supposed in earlier years of PHA research (Lundgren et al. 1964).

Many decades of PHA investigation engendered three functionally different types of PHB present in cells:

1. The most common, high molecular weight storage PHB (with more than 10^3^ 3-hydroxybutyrate residues), with the dominating role of a storage material
2. Oligo-PHB (low molecular weight PHB with approximately 100–200 units of 3-hydroxybutyrate
3. Conjugated PHB (“c-PHB”) consisting of approximately 30 or less units, covalently linked to proteins

The last two types occur in various organisms, suggesting a general task different from a function as storage materials (Jendrossek and Pfeiffer 2014). Furthermore, the diversity of storage-PHA producers (concerning number of genera and species), and among them the diversity in PHA content, chemical composition, and molecular weight (concerning its size distribution), attracts attention of both the scientific and the professional community.

Generally, the size of PHA-containing cells increases with increasing PHA content, paralleled by the changing of typically stretched ellipsoid cell shape to more spherical contours. PHA accumulation ends either when the carbon feed is stopped or for steric reasons when the granule’s surface gets shielded from polymer precursors by forming highly compact granule packages (Jurasek and Marchessault 2004). For *Cupriavidus necator*, older literature reports a typical number and diameter of PHA granules after termination of bacterial growth with 10 and 0.5 μm, respectively (Anderson and Dawes 1990; Ballard et al. 1987). A variety of enzymes and structural proteins plus corresponding regulation mechanisms trigger PHA biosynthesis and granule formation (Jendrossek and Pfeiffer 2014; Pfeiffer and Jendrossek 2012; Pötter and Steinbüchel 2005). Homogeneity of PHA in terms of molar mass distribution is impacted by size and size distribution of PHA granules and decisive for processing towards marketable bioplastic items (Bradel and Reichert 1993; Bugnicourt et al. 2014). Size of PHA granules, the intracellular mass fraction of PHA, and number and location of PHA granules in cells depend on various factors. Especially the type and activity of phasins as structural proteins with decisive impact on PHA granule formation and prevention of coalescence of PHA granules are strongly dictating the intracellular cell structure (Mezzina et al. 2015; Obeso et al. 2015; Pötter et al. 2002; Pötter et al. 2005; Rehm and Steinbüchel 1999; Ushimaru et al. 2015; York et al. 2002). The nutritional environment (Wang and Lee 1997; Marang et al. 2015) or the activity of PHA synthases (Sim et al. 1997) predominantly influences the intracellular mass fraction of PHA and PHA’s molar mass distribution. In this context, PHA synthase activity is triggered by external factors such as pH value, temperature, or substrate concentration; this activity determines molar masses (Rehm and Steinbüchel 1999) and even defines the intracellular granule location (Sudesh et al. 2002). Particularities of the microbial production strain additionally impact PHA granule formation and granule structure (Hauf et al. 2015; Hokamura et al. 2015; Zinn et al. 2001). From the technological point of view, larger PHA granules facilitate PHA recovery grace to their rather low density (Wang et al. 2014). Further, immobilizing target compounds at the granule’s surface by docking to granule-associated proteins enables designing functionalized micro/nano drug carriers (Grage et al. 2009).

Despite the extensive investigation of PHA, the mechanism of granule formation on the molecular and cellular level is not completely elucidated yet. Different models of granule formation were developed in the past. The first idea widely accepted by the scientific community envisioned that synthesis of PHB granules occurs randomly in the cellular space, resulting in the so-called micelle model of granule formation (Ellar et al. 1968; Gerngross and Martin 1995). This model implies that initiation of PHB granule formation could occur anywhere in the cytoplasm. It is based on the assumption that soluble PHB synthase (PhaC1) builds micelles in the cytoplasm, and, subsequently, the hydrophobic PHB chains progressively elongate inside the micelles (Stubbe and Tian 2003). Later on, based on the discovered binding of oligo-PHB in the cytoplasmic membrane, the “budding model” was developed, assuming the localization of PHB synthase (PhaC1) at the cytoplasmic membrane, together with the assumption of the initial granule formation taking place within or at the cytoplasmic membrane. After the initiation phase (or in the later stage of synthesis, respectively), it was supposed that the PHB granules get detached from the membrane. Only recently, it was found by in vivo experiments that, in the absence of PHB, PhaC1 is not freely distributed in the cytoplasma and, moreover, is not soluble. It was evidenced (Pfeiffer and Jendrossek 2013) that this enzyme is “nonhomogeneously distributed in the cytoplasm in the form of a presumably nucleoid-associated PhaM-PhaC1 complex” (PhaM is the granule-associated protein with phasin properties that can bind to PHB, DNA, and PhaC1 polymerase). In addition, this complex was reported as “constitutively expressed in *R. eutropha*,” linked to the nucleoid before PHB synthesis, and later bonded to the nucleoid and to the PHB granules (in statu nascendi). Based on the above-cited article and works of several laboratories (reviewed by Jendrossek and Pfeiffer 2014), the concepts of “budding and/or micelle models” were fine-tuned by introducing the “scaffold-type model” which assumes the attaching of PHB synthase to a scaffold molecule within the cell. The location of such scaffold molecules in the cell is assumed to be the determining factor for localization of PHB granules.

Experimental results that support one of these models are contradictory in the literature. For example, Jendrossek et al. (2007) reported that in *Caryophanon latum* DSM 484 and *Beijerinckia indica* DSM 1715, under conditions stimulating PHB synthesis at the very beginning of granule structuring, these carbonosomes are located close to the cytoplasmic membrane. These authors have investigated the granule formation in the first 20 min from induction of PHB formation, as well as in the range of 2 h after initiation of PHB synthesis. The localization of PHB granules was different for the two investigated organisms. In *B. indica*, it was found that 98 % of cells contained two PHB granules localized at one or two poles of the cells. The finding of three granules, two at the cell poles and one small in the middle of the cell, was rather rare, assuming that “a new granule can be formed at the site of septum formation” for cell division. Wahl et al. (2012) have explored the initiation of PHB granule synthesis under PHB permissive conditions in *Ralstonia eutropha* H16 and *R. eutropha* HF39 in the time range of 0–10, 20, 40, 60, 90, 180, and 240 min. They found that initial PHB granule synthesis was not randomly located but tightly bound with the nucleoid. In addition, the protein PhaM (the granule-associated protein with phasin properties that can bind to PHB, DNA, and PhaC1 polymerase) and some phasin proteins (i.e., PhaP5; Pfeiffer et al. 2011) were stressed in *R. eutropha H16* as important elements regulating “number, surface to volume ratio, subcellular localization and distribution of poly(3-hydroxybutyrate) to daughter cells” as well as the detachment of PHB granules from nucleoids. In the first hour after PHB synthesis initiation, a lot of cells are characterized by one or two PHB granules attached to the nucleoid. Within the first hour of growth, the granules were 100–300 nm in diameter. If cells harbored two PHB granules, these were dominantly located at opposite sides of the nucleoid. After more than 1 h of growth under conditions favoring PHB synthesis, number and size of the granules were increased (for the strain H16 up to 12 granules were evidenced, whereas the strain HF39 reached 1 to 4 granules). With growing number and diameter of accumulated PHB granules, the association of the granules with the nucleoid became less evident. Based on the above finding, the authors suggested the attachment of PHA granules to DNA as a general property of PHA-storing bacteria, which needs to be confirmed and further investigated.

Except early phases of PHB synthesis described above (initiation), some investigations were also performed for the late phases of cultivations; here, the behavior of fully developed PHB granule synthesis was examined by Vadlja (2015) and by Mravec et al. (2016) in order to study the possible sterical and biochemical hindrances when mass/volume fraction of PHB in the cell approaches its maximum. Mravec et al. (2016) have investigated *C. necator* H16 (formerly *R. eutropha* H16) in the period of 80 h of cultivation using confocal fluorescence microscopy (CFM) and scanning transmission electron microscopy (SEM). Authors have applied the determination of cell sizes and PHB granule sizes (i.e., width, length, and diameter) by “simple B/W pixel counting” image analysis using appropriate software. The estimation of volume fraction of granules/cells was based on the “stereology formula stating that, in systems with random morphology, the volume fraction and the area fraction in a random section through the system are approximately equal (Slouf et al. 2015).” In addition, the total volume of individual cells was calculated using a cell shape approaching a cylinder. Comparing the size measurements of cells and granules achieved by CFM and SEM, authors have concluded that results obtained by CFM are in agreement with SEM, but such results can be reached only by statistical analysis. This comparison was necessary because of possible differences in resulting PHB granule/cell sizes achieved in SEM (by ultra-thin slicing of objects at different sectional planes). Similar approach of PHB granule/cell size determination as followed by Mravec et al. (2016) was also performed by Vadlja (2015), who has analyzed the cells and granule size of continuously cultivated *C. necator* DSM 545 using transmission electron microscopic (TEM) images and pixel counting by ImageJ software. In this case, the cell/granule sizes were expressed as the area of objects present on binary converted TEM images. Concerning physiology of some PHA producers (triggering synthesis by P- and/or N-source depletion), for the long-term production, the two-stage continuous reactor systems are reasonable. In the latter case, catalytically active biomass is first produced under nutritionally balanced conditions and subsequently confronted with stress conditions like nutrient deprivation, thus shifting the carbon flux towards product formation. This system was further improved by the cascade of five continuously stirred bioreactors (5CSTR) implemented for the glucose-based, chemostat-type PHB production by *C. necator*. In such way, mimicking the characteristics of a tubular plug flow reactor, excellent PHB productivity, and homogeneity was displayed (Atlić et al. 2011). Using kinetic modeling, elementary flux modes (EFMs), and yield space analysis (YSA), a highly structured metabolic model was established to profoundly examine this process (Horvat et al. 2013; Lopar et al. 2013). The mentioned five-step cascade reactor system and predictive power of the above-cited models were used as a tool in the work by hand for studying PHB biosynthesis in the long-term cultivation of *C. necator*.

### Aim of the work

Mechanistic simulation of a typical *C. necator* cell during PHB accumulation was described before by Jurasek and Marchessault (2004), predictively describing enzymes and other proteins involved in carbonosome formation and stabilization, changes of the degree of polymerization, and some metabolic regulations. Jendrossek et al. (2007) have investigated the PHB granule formation at the very beginning of granule structuring (in the first 20 min from induction and 2 h after initiation). Wahl et al. (2012) have explored the initiation of PHB granule synthesis as well as its progress (every 20–30 min in the time range of 0–240 min) in *R. eutropha* H16 and *R. eutropha* HF39. Additionally, Mravec et al. (2016) worked on PHA formation by batch culture of *C. necator* H16 (formerly *R. eutropha* H16) in the period of 0–80 h of cultivation. In contrary, the behaviors and properties of PHA granules in the long-term, continuous, multistage cultivations (concerning cell to granule sizes, granule number, possible joining, and steric hindrance) were not investigated so far. In light of above-cited findings for PHA granule formation and monitoring, the work at hand is devoted to the investigation of storage PHB granules, concerning their genesis, their size progression, and their intracellular fate during long-term continuous cultivation of *C. necator*. This was done by far exceeding the maximal time ranges of all described cultivations found in the literature. Beyond these efforts, our work reflects de facto dimensions of microbial cells and PHA granules.

Therefore, the statistical distribution of cell and PHA granule size and number of granules per cell along the 5CSTR are addressed. Cells and PHA granules were directly analyzed by taking electron microscopic pictures and converting them to binary pictures. The evaluation of footprint areas from binary pictures for the PHA and non-PHA part of cells allowed the determination of minimal, maximal, and average size of cells and inclusions. By correlating these observations to experimentally determined kinetics and environmental conditions, our approach permits to draw conclusions on the performance of each individual stage of the 5CSTR. The trends observed along the cascade, combined with data previously obtained from mathematical modelling (Horvat et al. 2013, Lopar et al. 2013), will enable the future design of multistage PHA production facilities with even higher industrial potential.

## Materials and methods

### Microbial production strain

*C. necator* DSM 545 (formerly referred to as *Wautersia eutropha*, *R. eutropha*, *Alcaligenes eutrophus*, or *Hydrogenomonas eutropha*) was obtained from Deutsche Sammlung von Mikroorganismen und Zellkulturen GmbH (DSMZ), Germany. The strain was cultivated in minimal medium according to Küng (1982).

### Cultivation in the multistage bioreactor cascade

The chemostat continuous cultivation was carried out in a five-stage bioreactor cascade consisting of continuously stirred tank reactors (CSTRs) allocated from Infors, CH. All details of the cultivation process were reported previously (Atlić et al. 2011; Horvat et al. 2013; Lopar et al. 2013). Most importantly, the first CSTR, R1, was dedicated to balanced microbial growth to generate active biomass, R2 acted as transient CSTR to metabolize residual nitrogen source (NH_4_^+^), whereas CSTRs R3 to R5 were designated for PHA accumulation under nitrogen-free conditions. Carbon source (glucose) was added continuously to all CSTRs. Dilution rate *D* in the different stages amounted to 0.17 1/h for (R1) and 0.21 1/h for (R2), (R3), (R4), and (R5), respectively. Corresponding residence time *τ* in the different stages was as follows: 5.88 h for (R1) and 4.76 h for (R2), (R3), (R4), and (R5). Total *τ* of the system (cumulative) is 5.88 h (R1), 10.64 h (R2), 15.40 h (R3), 20.16 h (R4), and 24.92 h (R5).

#### Nota bene

Samples used for the investigations (traditional analysis and imaging) have been taken after 232 h of continuous cultivation, where the entire 5CSTR was in steady state.

### TEM pictures

Electron microscopic (EM) dark field imaging was done from ultra-thin cross sections of samples performed by microtome; they were stained by uranyl citrate plus lead acetate and entrapped in synthetic resin. Zeiss Ultra 55 raster electron microscope and a scanning transmission electron microscope (STEM) detector (in transmission) were used. Hence, the pictures constitute TEM pictures but were generated in SEM mode at lower acceleration voltage (30 kV). This work was carried out courtesy of FELMI-ZFE, Graz.

### Conversion of TEM pictures to binary pictures by ImageJ software

TEM pictures of microbial populations (from cascade reactors R1–R5) were analyzed by ImageJ software (Anonymous 1, 2014), an open source image processing program designed for scientific multidimensional images by the National Institute of Mental Health, Bethesda, MD, USA. Pictures were first converted in grayscale 8-bit format and thereafter to binary format. The scale was further calibrated (pixel-to-length) using the “set scale” option and linear ruler from TEM pictures. Contours of those cells located on the outer picture edges as well as those featuring suspiciously sharp cell or/and PHB edges were neglected. PHB granules received white coloration, well contrasted from the gray/black residual biomass. Assuming a direct proportionality between the imaged cell/granule area and the related cell/granule volume, binary pictures were used for counting of cells and granules, for the “cell by cell” measuring of whole cell sizes/areas (i.e., black and white fields) as well as for the determination of PHB granule sizes (white areas).

### Statistical analysis and related tools

Size of whole cells and PHB granules, expressed as the area of the footprints on binary EM pictures, characteristic variables (minimal, maximal, and average values of areas), and distributions of the variables were analyzed by the “STATISTICA” data analysis software system, version 8.0.

### Equations for calculation of specific growth and PHB synthesis rates

Analysis of biomass and PHB concentration in the individual reactor stages enabled the traditional calculation of specific growth rate *μ* (Eq. 1) and specific PHB production rate *π* (Eq. 3). Data obtained by cell and granule footprint area analysis of binary pictures were used for the novel approach to calculate *μ* (Eq. 2) and *π* (Eq. 4), respectively.

1 $$ \mu =\frac{\varDelta {X}_R}{\varDelta t}*\frac{1}{X_R}=\frac{X_{Rn}-{X}_{Rn-1}}{X_{Rn}}*{D}_{\left[R(n)\right]}\kern0.5em \left[{h}^{-1}\right] $$

2 $$ \begin{array}{cc}\hfill {\mu}_{\left[\mathrm{R}\left(\mathrm{n}\right)\right]}^{*}=\left(\frac{\overline{P}{(SP)}_{\left[\mathrm{R}(n)\right]}-\overline{P}{(SP)}_{\left[\mathrm{R}\left(n-1\right)\right]}}{\overline{P}{(SP)}_{\left[\mathrm{R}(n)\right]}}\right)*{D}_{\left[\mathrm{R}(n)\right]}\hfill & \hfill \left[{\mathrm{h}}^{-1}\right]\hfill \end{array} $$

3 $$ \begin{array}{cc}\hfill {\pi}_{S\left[R(n)\right]}=\frac{D_{\left[R(n)\right]}*\left[c{(PHB)}_{\left[R(n)\right]}-c{(PHB)}_{\left[R\left(n-1\right)\right]}\right]}{X_R}\hfill & \hfill \left[\frac{g_{(PHB)}}{g_{(RBM)}h}\right]\hfill \end{array} $$

4 $$ \begin{array}{cc}\hfill {\pi}_{S\left[R(n)\right]}^{*}=\frac{D_{\left[R(n)\right]}*\left[\overline{P}{(PHB)}_{\left[R(n)\right]}-\overline{P}{(PHB)}_{\left[R\left(n-1\right)\right]}\right]}{\overline{P}{(SP)}_{\left[R(n)\right]}}\hfill & \hfill \left[\frac{{\left(\mu m\right)}_{PHB}^2}{{\left(\mu m\right)}_{SP}^2*h}\right]\hfill \end{array} $$

where *X*_R_ is concentration of residual biomass in individual bioreactor [g/L]; *D*_[R(*n*)]_ is dilution rate in *n*th cascade reactor [h^−1^]; R(*n*) is bioreactor designation (*n* = 1–5); *μ* is specific growth rate [h^−1^]; c(PHB) is concentration of PHB in individual bioreactor [g/L]; $\overset{-}{\mathrm{P}}(PHB)$ is average area of PHB granules on binary pictures in square micrometer; $\overset{-}{\mathrm{P}}(SP)$ is average size of whole cells on binary pictures [μm^2^]; *π* is specific PHB production rate [g g^−1^ L^−1^]; and asterisk indicates the variables calculated by data from ImageJ analysis.

## Results

### Results of TEM picture analysis and related binary conversions by ImageJ software

**Fig. 1:**
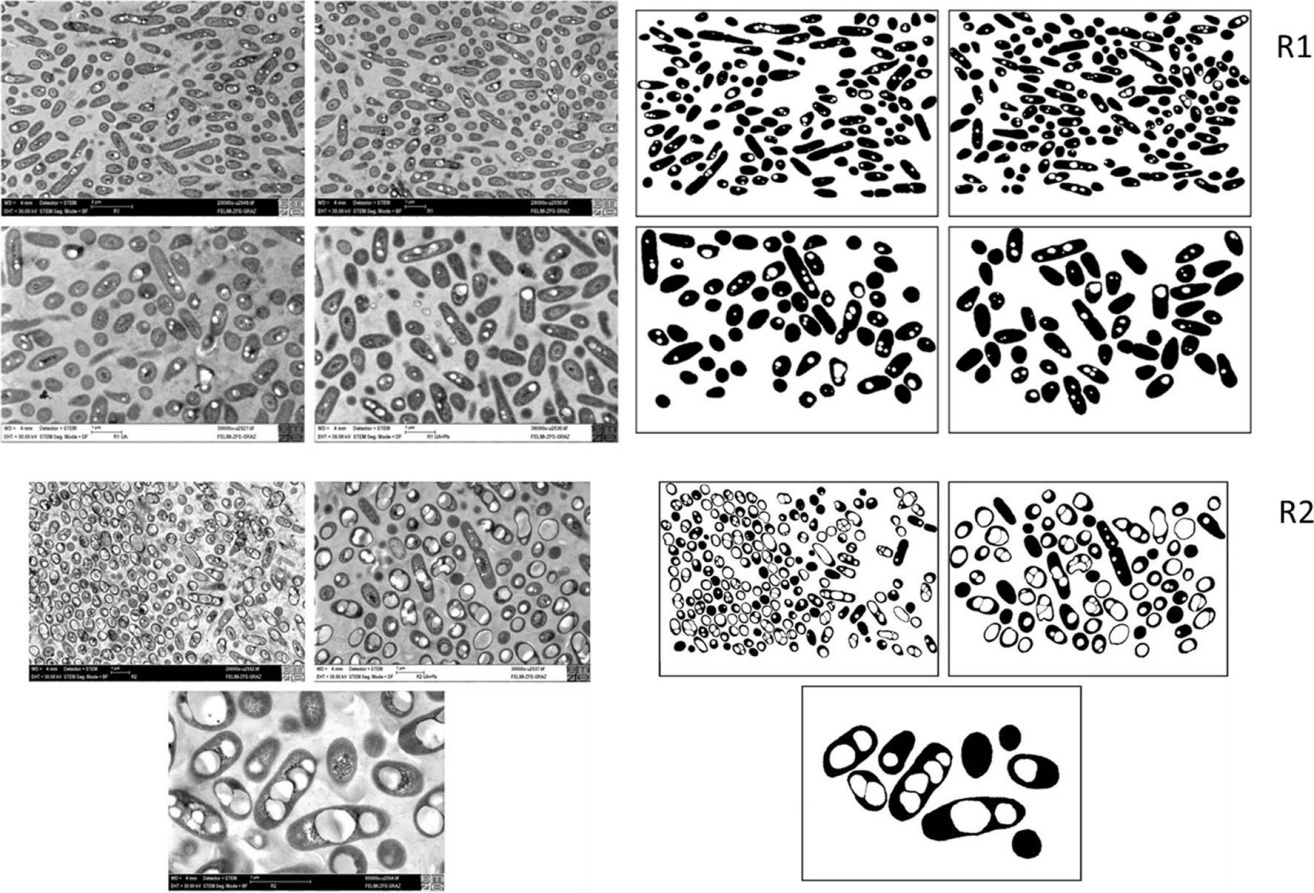
TEM pictures of *C. necator* DSM 545 culture taken from first (*R1*) and second (*R2*) cascade reactor. R1 (*upper left group of four pictures*), R2 (*lower left group of three pictures*). Binary versions of the same photos generated by ImageJ software are placed on the right side. R1 (*upper right group of four pictures*), R2 (*bottom left group of three pictures*). The magnifications were ×20,000 and ×30,000 (R1 culture) and ×20,000, ×30,000, and ×65,000 (R2 culture). The corresponding dilution rates *D* were 0.17 h^−1^ (R1) and 0.21 h^−1^ (R2), with residence times *τ* of 5.88 h (R1), 4.76 h (R2), and 10.64 h (the total residence time for R1 + R2)

Each stage of the 5CSTR was functionally assigned according to its dedicated individual role. Cell growth (biomass formation) occurred in R1 and R2, with R1 assigned to efficient biomass growth and R2 acting as “transient reactor,” dedicated to ultimate depletion of nitrogen source. R3, R4, and R5 were dedicated to PHB accumulation without any concurrent biomass formation. Results presented below are grouped in accordance to these individual functions. Results of TEM imaging of samples taken from R1 are shown as upper left group of four pictures in Fig. 1; here, cells are depicted in magnifications ×20,000 and ×30,000, respectively. The low mass fraction of PHA in biomass (*m* = 0.07 g/g) is visible as bright inclusions. Figure 1 also shows related binary processed versions as obtained by the ImageJ software tool (upper right group of four pictures). Here, black parts of the cells indicate the non-PHA part of biomass, whereas PHB inclusions are clearly contrasted by their white coloration.

**Fig. 2:**
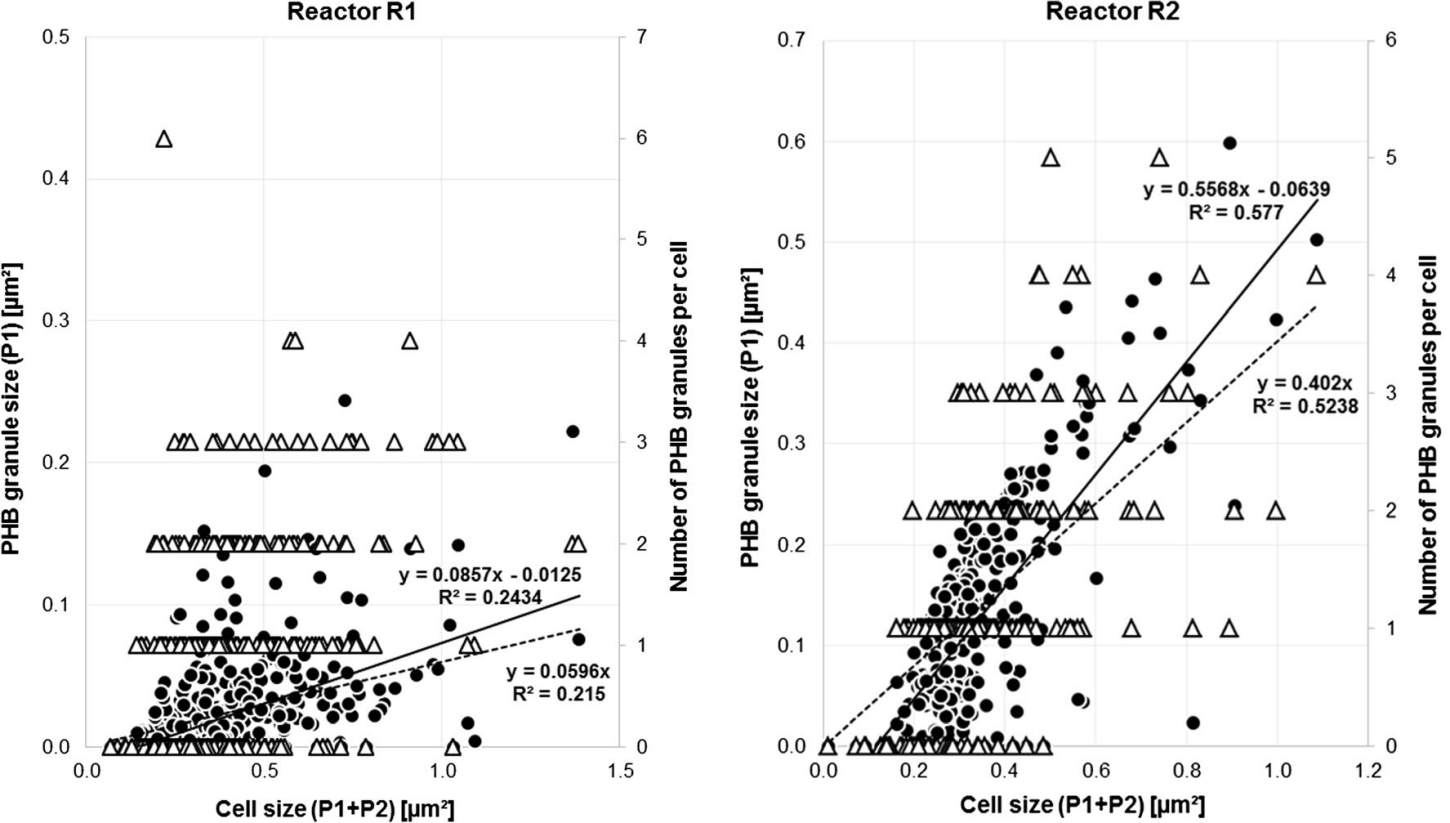
The dependency of PHB granule sizes (*black circle*) and the number of PHB granules per cell (*white up-pointing triangle*) on the whole cell size in a population of *C. necator* DSM 545 from reactor R1 (*left picture*) and R2 (*right picture*) of the five-step reactor cascade. Both *straight lines* are the *best fit* lines for relation of granule to cell size, drawn with (*broken line*) and without (*straight line*) forcing to the origin of the coordinate system, respectively. The “origin included” *straight line* refers to the assumption that PHB granules grow in parallel with cellular growth. Data were achieved by use of ImageJ area analysis of binary rearranged SEM photos

Table S1 provides a compilation of the obtained measuring results necessary for the statistical analysis of “footprint areas” of whole cells (PHB + residual cell part), PHB granules, and their ratio. This encompasses the total cell area, minimal, average, and maximal cell and PHB granule sizes, respectively, and minimal, average, and maximal PHB-free cell parts. Further, Table S1 presents the number of PHB granules in cells, showing that a maximum of six granules was detected per individual cell. These data were used for graphical analysis by plotting the area of PHB granules [μm^2^] and the number of granules in dependence of the cell area [μm^2^] (Fig. 2, left) in order to obtain the mathematical relation between examined variables.

Results of TEM imaging of samples from R2 are shown in Fig. 1 (bottom left group of three pictures), where cells are depicted in magnifications ×20,000, ×30,000, and ×65,000, respectively. The accumulated PHB (*m* = 0.48 g/g) is visible as bright inclusions. In binary versions of these pictures (group of three pictures in the bottom right corner), black parts of the cells indicate the non-PHA part of biomass, whereas PHA inclusions are clearly contrasted by their white coloration.

Table S2 provides a compilation of results related to samples from reactor R2. As in the previous case (R1), these data were used for graphic analysis by plotting the area of PHA granules [μm^2^] and the number of granules per cell in dependence on the total cell shape area [μm^2^] originated from binary converted photos (Fig. 2, right picture).

**Fig. 3:**
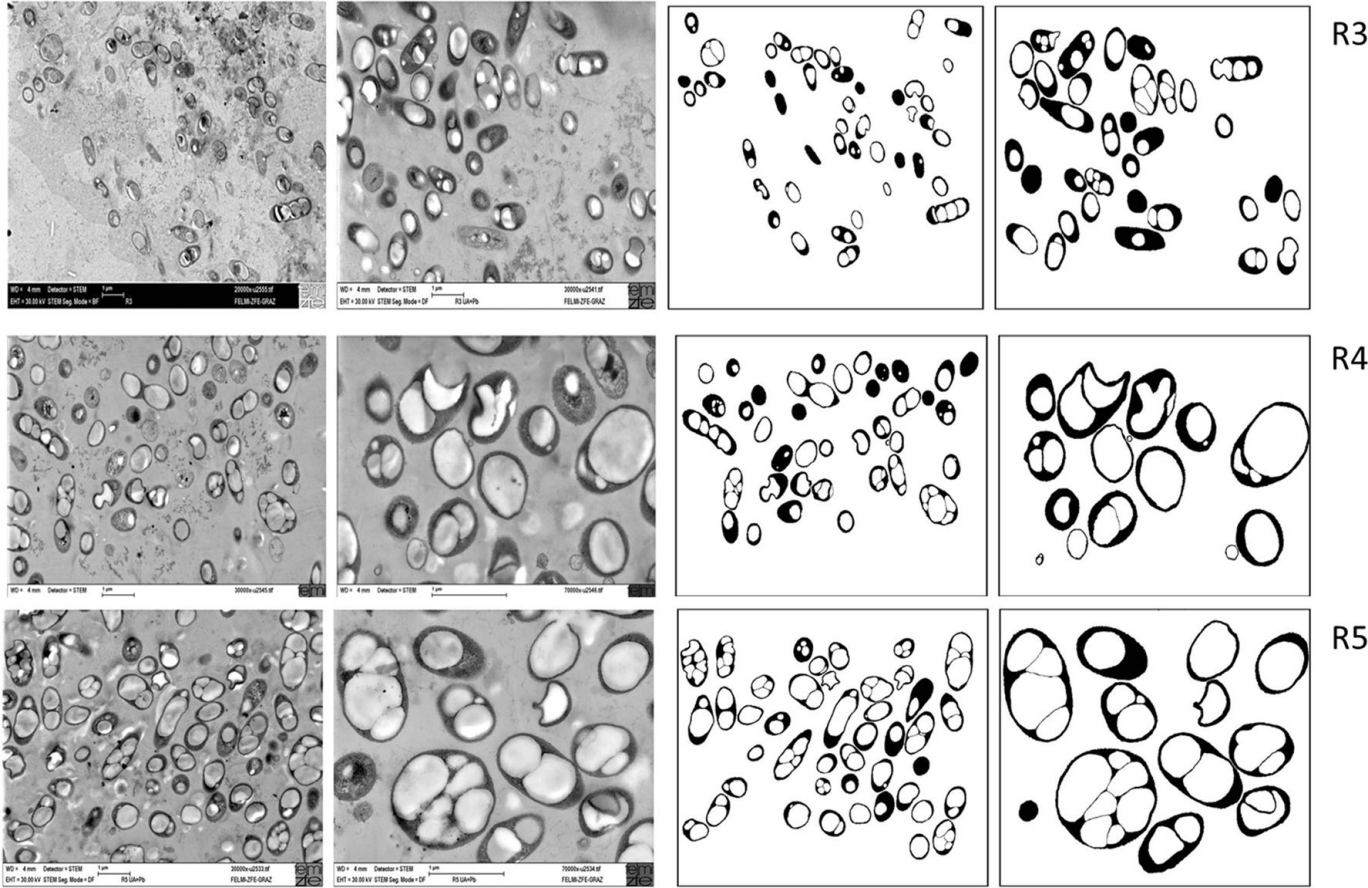
*Upper row*: TEM pictures of *C. necator* DSM 545 culture taken from third reactor (R3) of the reactor cascade (*left two pictures*) and binary versions of the same photos generated by ImageJ software (*right two pictures*). The related magnifications applied were ×20,000 and ×30,000. In this reactor, the dilution rate *D* and related residence time *τ* were 0.21 h^−1^ and 4.76 h, respectively, the total *τ* in the system (R1 + R2 + R3) amounted to 15.40 h. *Middle row*: TEM pictures of *C. necator* DSM 545 culture originated from the fourth reactor (R4) of the reactor cascade (*left two pictures*) and binary versions of these photos generated by ImageJ software (*right two pictures*). The magnifications applied were ×30,000 and ×70,000. In this reactor, the dilution rate *D* and related residence time *τ* were 0.21 h^−1^ and 4.76 h respectively, and the total residence time of the system (R1 + R2 + R3 + R4) amounted to 20.16 h. *Bottom row*: TEM pictures of *C. necator* DSM 545 from fifth reactor (R5) of the reactor cascade (*left two pictures*) with related binary versions of these photos generated by ImageJ software (*right two pictures*). The magnifications applied were ×30,000 and ×70,000. In this reactor, the dilution rate *D* and related residence time *τ* were 0.21 h^−1^ and 4.76 h, respectively, and the total *τ* in the system (R1 + R2 + R3 + R4 + R5) was 24.92 h

Results of TEM imaging of samples from R3 to R5 and related binary versions are shown in Fig. 3 (R3, upper row; R4, central row; and R5, bottom row). Here, cells were depicted in magnifications ×20,000 and ×30,000, respectively. In R3, *m* accounted to 0.55 g/g.

**Fig. 4:**
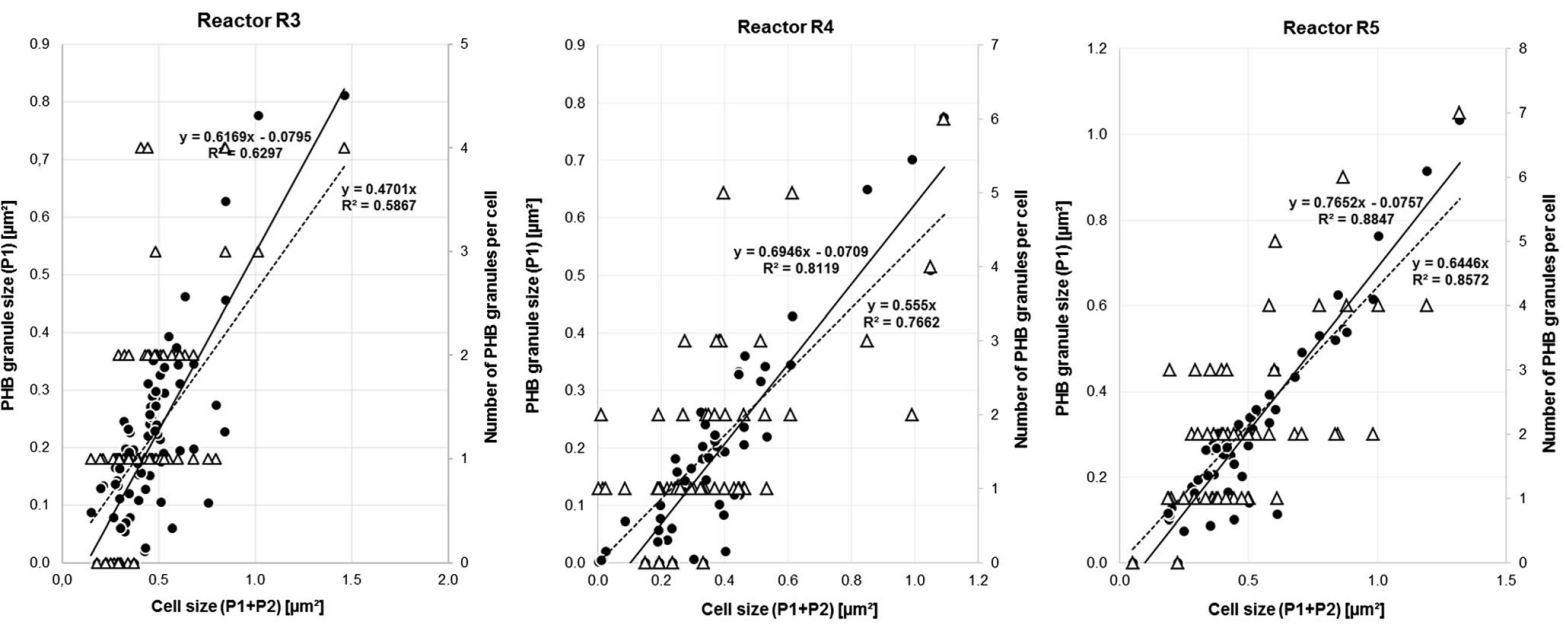
The dependency of PHB granule sizes (*black circle*) and the number of PHB granules per cell (*white up-pointing triangle*) on the whole cell size in population of *C. necator* DSM 545 from reactors R3 (*left picture*), R4 (*picture in the middle*), and R5 (*right picture*) of the five-step reactor cascade. Both *straight lines* are the best fitting lines for relation of granule to cell size, drawn with (*broken line*) and without (*straight line*) forcing to the origin of the coordinate system, respectively. The “origin included” *straight line* refers to the assumption that PHB granules grow in parallel with cellular growth. Data were achieved by use of ImageJ area analysis of binary rearranged SEM photos

Statistical analysis of footprint areas of whole cells and PHB granules was performed using the data presented in Table S3. These results encompass the binary imaged total cell areas, minimal, average, and maximal cell and PHA granule sizes, minimal, average, and maximal PHB-free cell areas, as well as the number of granules per cell. In this stage of the cascade, a maximum of four granules per cell was detected. Graphic analysis of dependency of PHB granule sizes [μm^2^] and the number of granules per individual cell on the whole cell size is presented in Fig. 4 (left picture).

In Fig. 3 (central row, left), PHB granules present in R4 (*m* = 0.65 g/g) are visible as bright inclusions. Results of cells and PHB granule sizes achieved by ImageJ software are provided in Fig. 4 (middle picture) and Table S4. They encompass results for R4 for the same parameters as Tables S1–S3 show for R1–R3.

Results of TEM imaging for R5 are shown in Fig. 3 (third row left) together with related binary versions (third row, right). These samples were depicted in magnifications ×20,000 and ×30,000, respectively. For samples originating from this reactor, *m* amounted to 0.69 g/g.

Table S5 collects results of whole cells and PHB granule size determinations, respectively, achieved by binary imaging. Further, it analyzes the number of PHB granules in the individual cells, showing that maximum seven granules per individual cell were detected in R5. These data were used for graphic analysis by plotting PHA granule sizes (expressed as area [μm^2^]) and the number of granules per cell in dependence of the whole cell size (Fig. 4, right picture).

### Statistical analysis of whole cells and PHB granule sizes (shape areas)

**Fig. 5:**
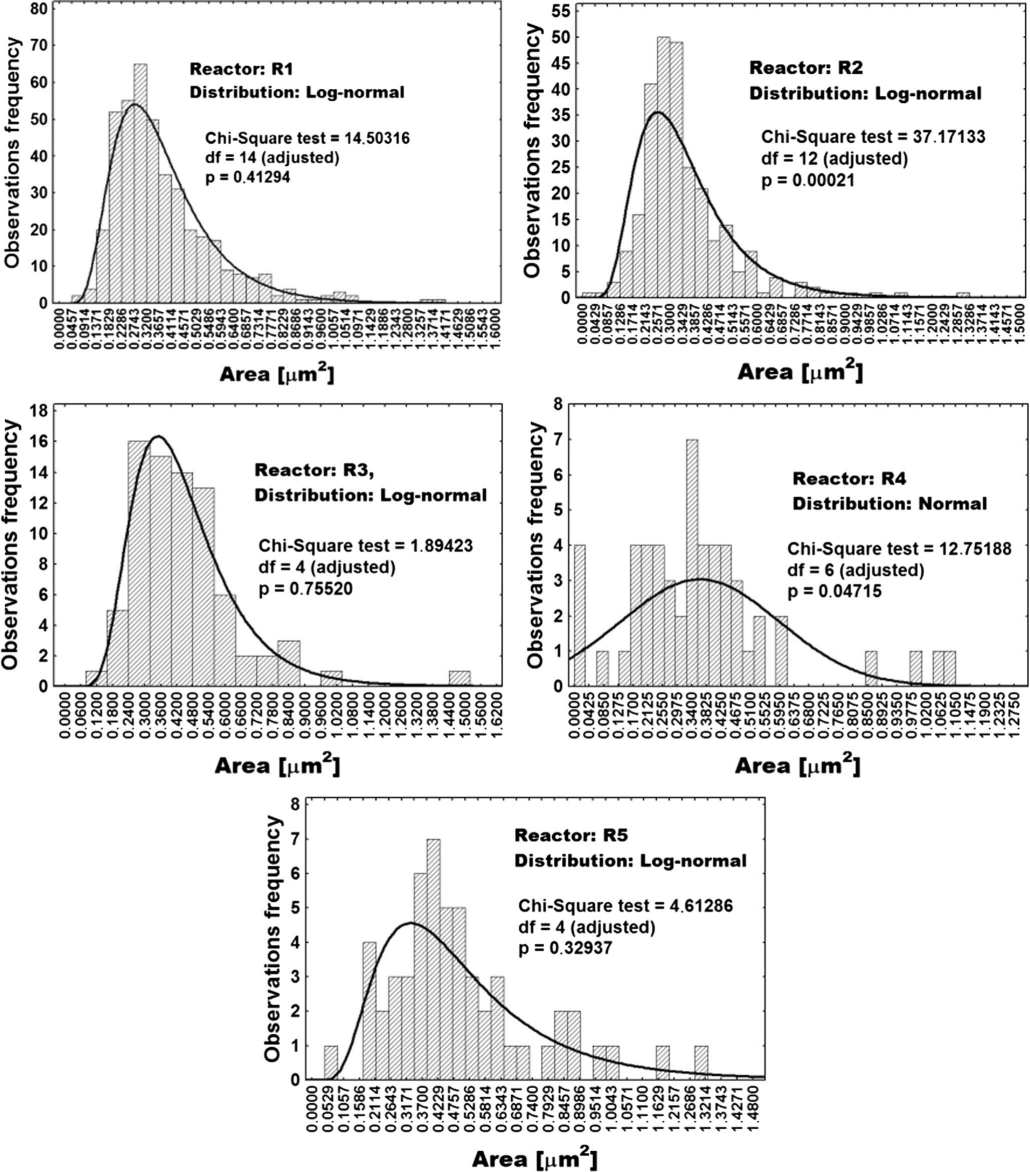
Distribution of whole cell sizes in the bioreactor cascade estimated by ImageJ software area analysis tool and binary converted SEM photos. Legend: *df* = degrees of freedom; *p* = *p* value

Comparative presentation of whole cell size distributions for all five reactors in the cascade is provided in Fig. 5.

**Fig. 6:**
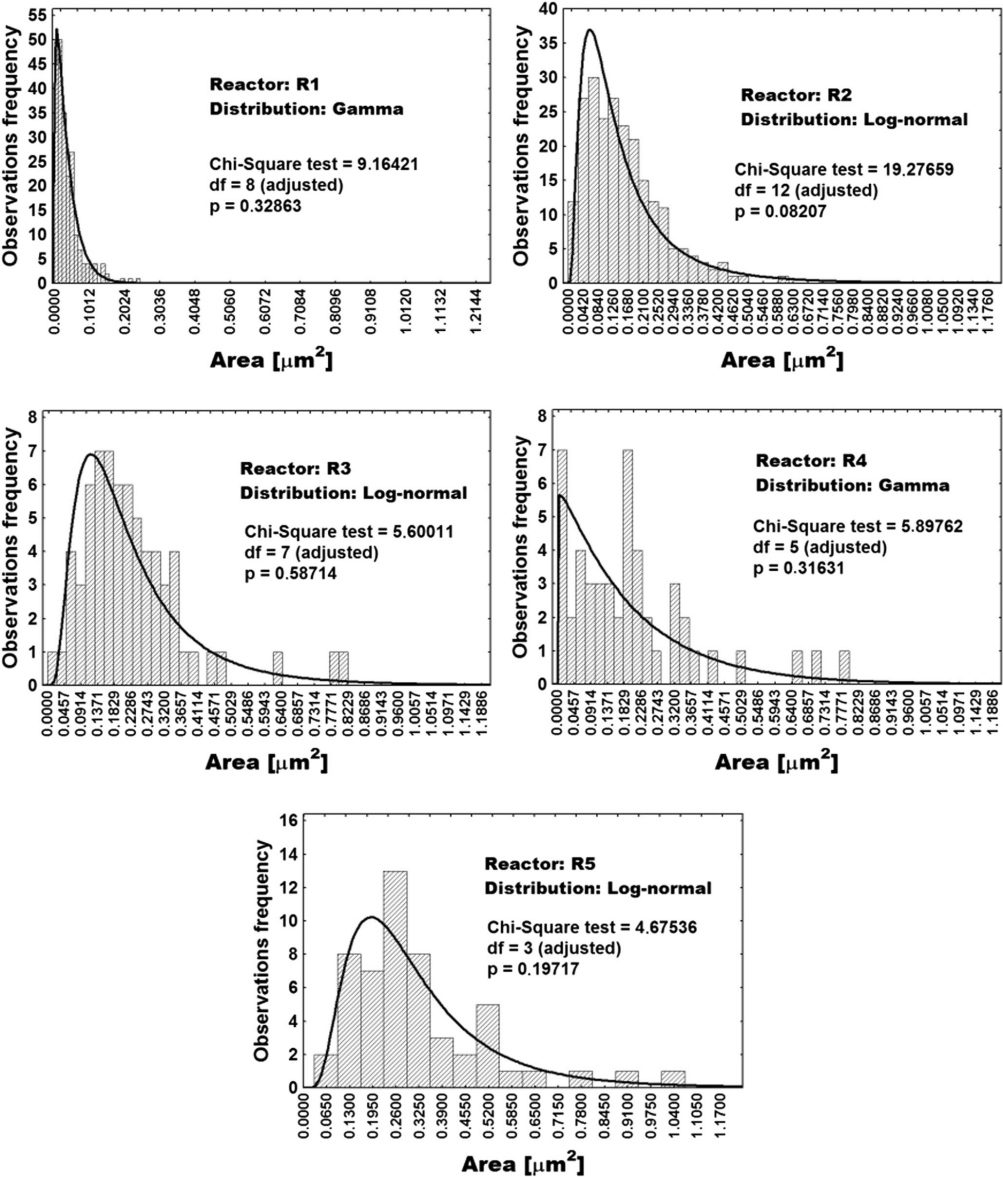
Distribution of PHB granule sizes in bioreactor cascade estimated by ImageJ software area analysis tool and binary converted SEM photos. Legend**:** *df* = degrees of freedom; *p* = *p* value

Comparative presentation of distributions of PHB granule sizes for all five reactors in the cascade is provided in Fig. 6.

### Comparative analysis of 5CSTR system

**Fig. 7:**
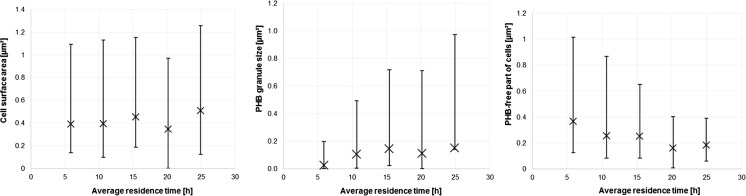
Dependence of minimal (*low points*), maximal (*high points*), and average values (*multiplication sign*) of whole cell sizes (*left picture*), PHB granule sizes (*middle picture*), and PHB-free part of cells (*right picture*) of *C. necator* DSM 545 on *τ* in five-step continuous reactor system

**Fig. 8:**
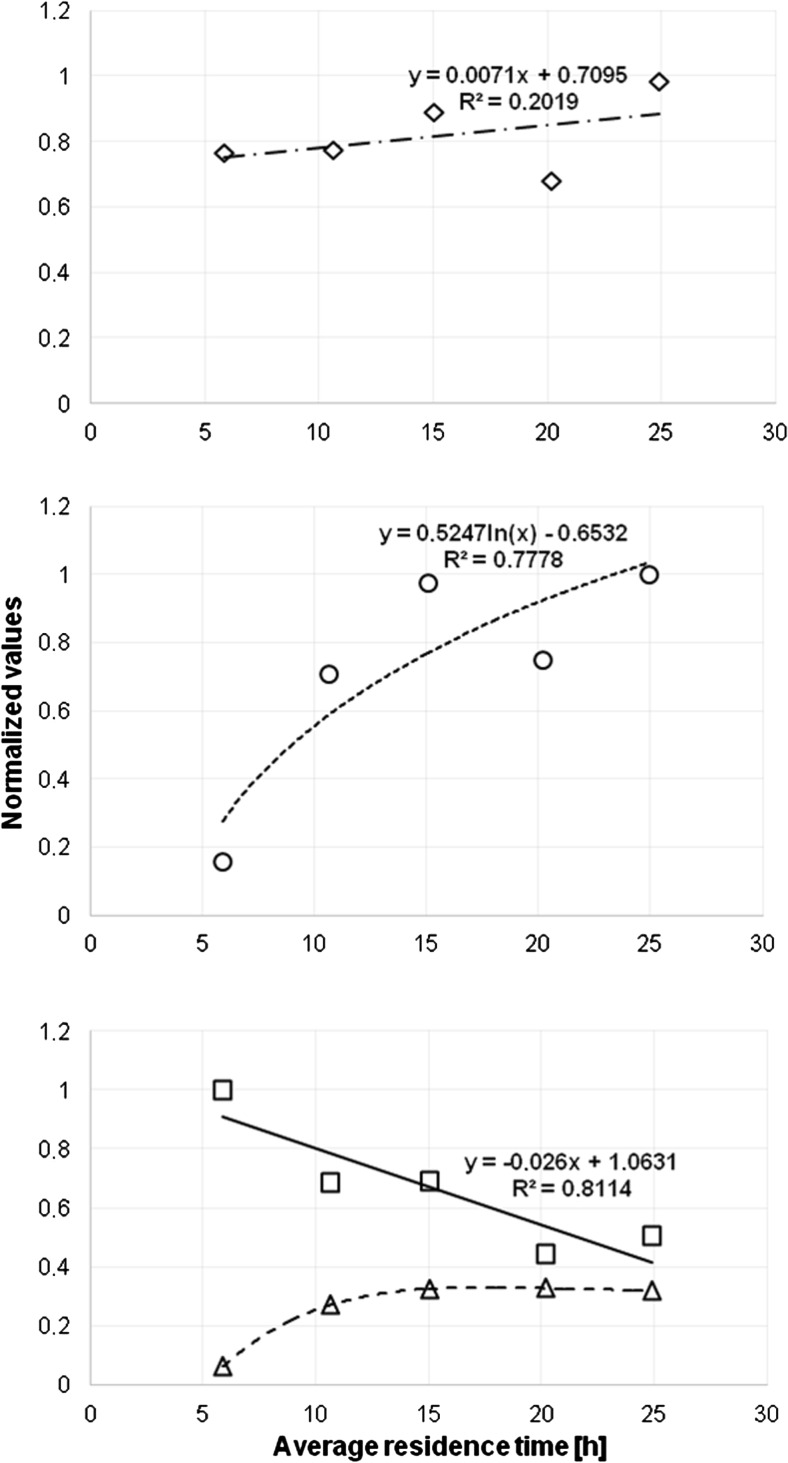
The change of normalized averaged values of whole cell sizes (*diamond*), PHB granules (*white circle*), PHB-free part of cells (*white square*), and ratio PHB/whole cell size (*white up-pointing traingle*) during cultivation of *C. necator* DSM 545 in five-step continuous reactor system. The maximal values of related variables are set to 1

Minimal, maximal, and average values of whole cells (left picture), PHB granules (central picture). and residual biomass sizes (right picture) in dependency on *τ* along the 5CSTR cultivation are presented in Fig. 7. The sizes are presented as related shape areas, estimated by ImageJ software using binary converted TEM photos. Normalized values of the abovementioned variables are presented in Fig. 8 [whole cell sizes (upper picture, rhombs), PHB granules (second picture, dots), PHB-free part of cells (third picture, squares), and ratio PHB/whole cell size (lower picture, triangles)].

**Fig. 9:**
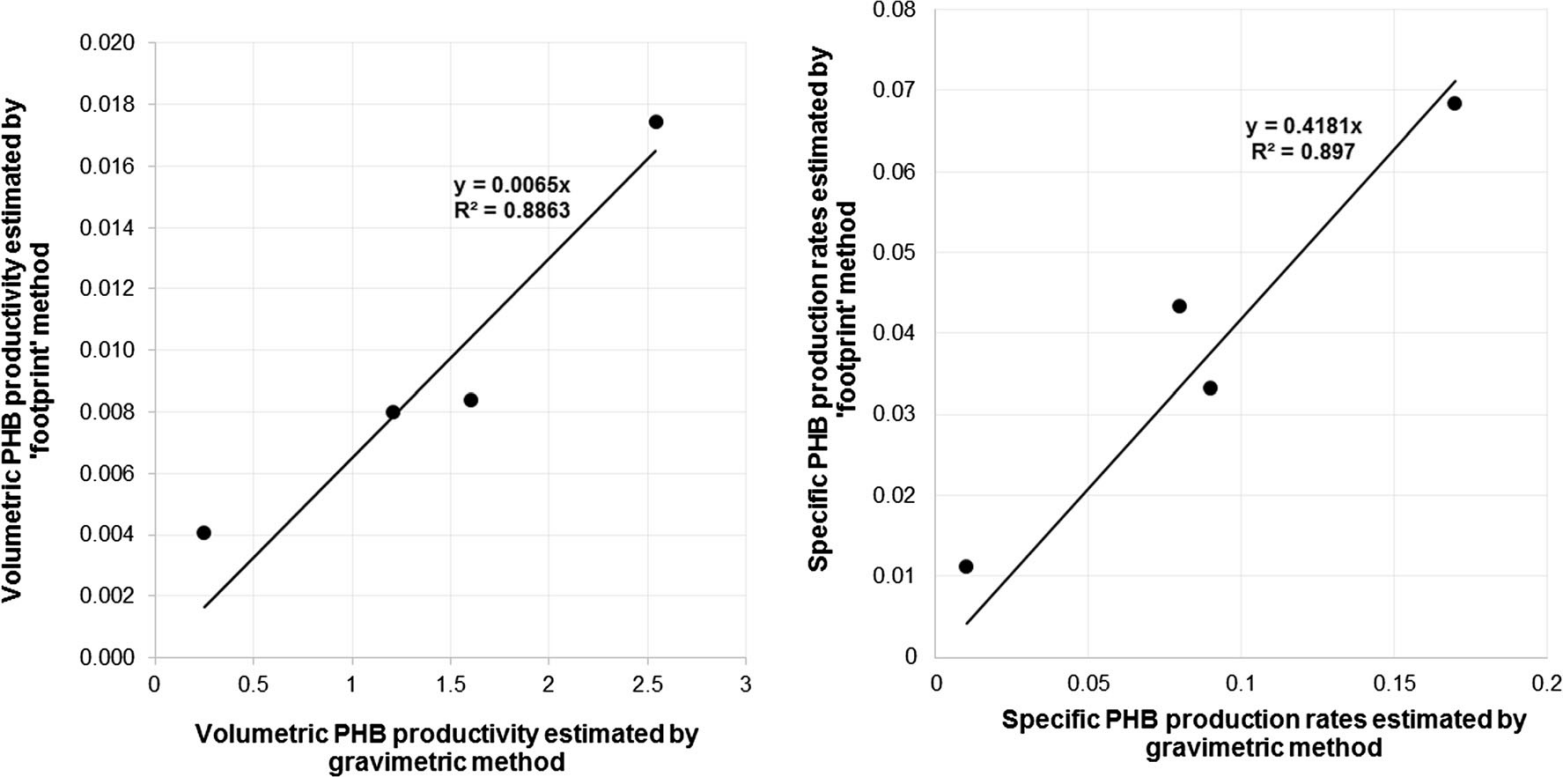
*Left picture*: The relation of results for volumetric PHB productivities (*q*) estimated by gravimetric and by “footprint” method. *Right picture*: The relation of results for specific PHB production rates (*π*) estimated by gravimetric and by “footprint” method

The agreement of data for volumetric PHB productivity (*q*) and specific PHB production rates (*π*) estimated by classic gravimetric method with the values of same variables (q*, π*) estimated by footprint area analysis is presented in Fig. 9 and Table 1.

**Table 1:** Comparative data for specific growth rates and specific production rates of PHB for *C. necator* DSM 545 cultivated in the five-step continuous reactor system

Reactor in cascade		R1	R2	R3	R4	R5
Dilution rate *D*	[h^−1^]	0.17	0.21	0.21	0.21	0.21
Residence time *τ* (related to each reactor)	[h]	5.88	4.76	4.76	4.76	4.76
Total residence time along the cascade	[h]	5.88	10.64	15.40	20.16	24.92
Specific growth rate (estimated gravimetrically)	[h^−1^]	0.17^a^	0^a^	0^a^	0^a^	0^a^
Specific growth rate (estimated by “footprint” method)	[h^−1^]	0.17	−0.09 (≈0)	0.00	−0.12 (≈0)	0.03 (≈0)
Specific PHB production rate (estimated gravimetrically)	$\frac{{\mathrm{g}}_{(PHB)}}{{\mathrm{g}}_{(RBM)}\times \mathrm{h}}$	0.01^a^	0.17^a^	0.09^a^	0.12^a^	0.08^a^
Specific PHB production rate (estimated by “footprint” method)	$\frac{{\left(\upmu \mathrm{m}\right)}_{PHB}^2}{{\left(\upmu \mathrm{m}\right)}_{SP}^2\times \mathrm{h}}$	0.0111	0.0684	0.0332	0.0441	0.0434
Average number of PHB granules per cell		1.59	1.72	1.56	1.79	1.81

^a^Values estimated gravimetrically

The influence of *τ* on the fraction of those cells containing PHB granules among the whole cell population as well as on the cell fractions containing one, two, or three granules per cell, respectively, is presented in the supplementary file Fig. S1.

## Discussion

Achieved results will be first discussed separately for each reactor, and thereafter, they will be compared and interconnected.

### CSTR R1

R1 of the 5CSTR cascade is devoted to balanced biomass growth and cell division. *C. necator* DSM 545 is characterized by considerable PHB synthesis when exposed to excess carbon source even without simultaneous nitrogen and/or phosphate limitation. TEM pictures of the culture from R1 were successfully processed by ImageJ software to facilitate cell and granule counting and to enable distinguishing and measuring of footprint areas of cells and PHB granules. Digital imaging turned out to be a convenient method to convert to binary (black/white) images (Fig. 1, upper left and related right group of four pictures). Applying this procedure, all blurred silhouettes of cells that were not in focus of the microscope were removed in order to correctly interpret the ImageJ results: only those objects with sharply outlined edges of cells and the corresponding PHB granules were taken into account. In Fig. 1 (upper left group of four pictures), some cells are visible that are in late stage of cell division, as well as pronounced elongated cells without clear evidence of septum formation. Both of these cell types were counted as one individual cell, until the clear separation of daughter cell from mother cell occurs. It can be seen that in all these cells, PHB granules are not bigger and not higher in number per cell than in average. For typically elongated cells without clear evidence of a septum, it was difficult to state about their “status,” e.g., are these cells in division process with (for unknown reason) stopped septum synthesis or do these cells (trichomes!) have interrupted nucleoid division, as described by Jendrossek et al. (2007) and Wahl et al. (2012). Elongated cells with higher number of PHB granules than average but with significantly bigger PHB granules than presented in Fig. 1 were evidenced for cultivation of *C. necator* H16 by Mravec et al. (2016). It is not clear if this difference between results of Mravec et al. (2016) and the work at hand results from intrinsic strain properties. Here, it is necessary to mention that Mravec et al. (2016) hypothesize that there is no evidence for a direct control between cell size and number of PHA granules and suppose that “both PHA biosynthesis and cell length are influenced by the same factors (e.g. nutrient limitation).”

As the ImageJ software offers an opportunity for the determination of minimal, maximal, and average size of sharply edged objects of whole cell populations, it was used for statistical analysis of imaged footprint areas of whole cells, PHB granules, and the number of granules per cell (Table S1). In this reactor, 55.8 % of cells contained PHB granules, 30.9 % were equipped with one granule, and 17.3 % with two granules. It was very interesting to analyze the position of granules for the mentioned two groups of cells. When two small granules were found, they were dominantly located close to the nucleoid (frequently on opposite sites and rarely on the same side). This is in accordance with findings of Wahl et al. (2012) for *R. eutropha* H16 but different to results for *B. indica* achieved by Jendrossek et al. (2007) where the granules were located at the cell poles. In the same analysis, it was observed that those cells harboring one PHB granule are characterized by relatively large granules, presumably because 5.88 h of *τ* for R1 is long enough to provide the progression of PHB biosynthesis. These granules were large enough to be at the same time instantaneously in the vicinity of the nucleoid as well as close to the plasma membrane, so a connection with the plasma membrane cannot be deduced.

Resulting values of interdependence of cell/granule sizes for R1 and related distributions are presented in Figs. 2, 5, and 6. At first, it was intended to find out if the size of granules correlates to the size of cells and if the number of granules per cell depends on the cell size. Figure 2 (left picture) presents results obtained by correlating the PHB granule size to the whole cell size. Here, it is well visible that a clear correlation only exits for relatively small cells (<0.8 μm^2^), whereas with increasing cell size, scattering of results enormously increases. This is valid for both applied best fit straight lines (with and without forcing to the origin of the coordinate system). The “origin included” straight line refers to the assumption that PHB granules grow in parallel with cellular growth, whereas the “origin excluded” straight line hypothesizes that a certain cell size must be achieved before PHB synthesis starts. Because of the only minor difference between the Pearson’s correlation coefficients obtained for these two lines (*R* ^2^ = 0.2434 and 0.215, respectively), it is not easy to conclude which of the two assumptions more realistically reflects the real situation. In addition, the rather low values of these coefficients indicate the high heterogeneity (in terms of the ratio of PHB granule size to cell size) of the bacterial population growing under nutritionally balanced conditions. Furthermore, the number of individual granules per cell cannot be related to the cell size for the balanced cultivation condition present in R1. Here, the number of granules per cell rarely exceeds the value of 3. Cells without PHB, as well as with one, two, or three granules, are practically uniformly distributed among the whole size range of cells (Fig. 2, left picture, triangle symbols).

### CSTR R2

R2 acts as a “transient reactor” where the nitrogen source added to R1 gets finally depleted. Therefore, some cellular growth is still possible in R2, but the cells undergo a considerable change regarding their intracellular metabolic fluxes from biomass synthesis to PHB production. Again, TEM images of the bacterial population in R2 (Fig. 1, lower group of three pictures, left) were processed to binary images (Fig. 1, lower group of three pictures, right). In contrast to R1, both cell types, i.e., long cells with relatively small PHB granules and long cells with PHB granules close to average dimension, are present. The latter cell/granule type was also found by other authorships, e.g., Jendrossek et al. (2007), Wahl et al. (2012), and Mravec et al. (2016)). Similar to R1, results of measuring footprint areas of whole cells and PHB granules (Table S2) were used for statistical analysis. Sufficiently sharp and clear binary images were advantageous to distinguish between PHB and the residual cell fraction. Figure 2 (right picture) shows that the number of PHB granules per cell (i.e., 0–3) is homogenously distributed among the whole range of cell sizes, but the footprint area of PHB granules, if compared to the footprint area of whole cells, was significantly enlarged (Fig. 1). Less variance of results for the interdependence of PHB granule areas and related cell areas in R2 (Fig. 2, right picture) is observed than in R1 (Fig. 2, left picture). This is reflected by the values of the Pearson’s correlation coefficients obtained for the straight lines (*R* ^2^ = 0.5238 if including the origin of coordinate system and *R* ^2^ = 0.5770 without inclusion of the origin, respectively). Although at a first glance an interdependence of the mentioned variables seems to be obvious, the relatively low values of both correlation coefficients warn for vigilance. However, based on Fig. 2 (right picture), it can be concluded that PHB granules are exclusively visible in such cells of footprint areas exceeding 0.2 μm^2^.

### CSTR R3

R3 is the first reactor along the cascade where no more growth of cells, but intensive PHB synthesis is expected. TEM images of the bacterial population from reactor R3 (Fig. 3, upper row, two pictures at the left) were transformed to binary images (Fig. 3, upper row, two pictures right) and statistically analyzed for footprint areas of whole cells and PHB granules (Table S3). In R3, the footprint area of PHB granules is already dominant over the footprint area of the PHB-free cell fraction. Here, it is striking that the number of elongated cells decreases if compared with R1 and R2; the cell shape becomes more ellipsoidal. Results presented in Fig. 4 (left picture) and Table S3 indicate that among the investigated cell size range, cells with zero or three PHB granules vanish in favor of cells harboring one or two granules (Fig. S1). This can be interpreted by the assumption that with increasing *τ*, those cells which in R1 and R2 were still PHB-free start to accumulate granules in R3, and those with three granules disappear because of steric hindrance of granules; here, granules seem to merge in only one carbonosomal space. If compared to the outcomes of R2, the variance of results for correlation of PHB size and cell size is slightly lower in R2, with somewhat higher Pearson’s correlation coefficients in R3, i.e., *R* ^2^ = 0.5867 with the origin included and *R* ^2^ = 0.6297 with the origin excluded, respectively. In addition, similar to the findings for R2, it is remarkable that granules are predominantly present in cells of footprint areas exceeding 0.2 μm^2^.

### CSTR R4

TEM images of bacteria collected from R4 (Fig. 3, central row, two left pictures) were converted to binary formats (Fig. 3, central row, two right pictures). Statistical analysis was performed as described for R1–R3 (Table S4 and Fig. 4, central picture). These illustrations clearly show that the majority of cells contain one single big granule (52.3 % of all cells, 56.1 % of the individuals containing granules); most other cells contain two granules (24.4 % of the whole population), whereas cells with zero or three granules are rare. If compared with other reactors, the scattering of results related to dependence of PHB footprint area on the whole cell area (Fig. 4, central picture) is significantly lower, as indicated by Pearson’s correlation coefficients *R* ^2^ = 0.7662, if including the origin point of coordinative axes, and *R* ^2^ = 0.8119, if excluding the origin. Also for R4, it turned out that the majority of cells containing PHB granules have a footprint area exceeding 0.2 μm^2^.

### CSTR R5

By analyzing the results presented in Fig. 3 (bottom row), Fig. 4 (right picture), and Table S5, it can be concluded that the number of PHB granules in the cells in most cases does not exceed the value of two. Notably, the footprint area of PHB granules, if compared to the whole cell’s area, is significantly higher than in the previous reactors R1–R4. The scattering of results presented in Fig. 4 (right picture) by correlation coefficients of *R* ^2^ = 0.8572 (origin point included) and *R* ^2^ = 0.8847 (origin excluded) is significantly lower than in all other reactors, indicating the high correlation between the two area types (PHB and PHB-free area).

### Comparative analysis of results for all reactors of the five-stage CSTR system

Statistical distribution of cell size (expressed as the footprint area of cells present in the 5CSTR devoted to production of PHB by *C. necator* in binary images) is presented in Fig. 5. The same was accomplished for PHB granule sizes (Fig. 6). Minimal, maximal, and average values of whole cell footprint areas as variables dependent on *τ* in the 5CSTR are shown in Fig. 7 (left picture). The same was accomplished for PHB granule areas (Fig. 7, central picture) and for the PHB-free parts of the cells (Fig. 7, right picture).

Concerning statistical distributions of cell sizes (Fig. 5), it can be concluded that the shape of distribution functions is fluctuating along the 5CSTR. The range of footprint areas of whole cells is 0.02–1.5 μm^2^. This variable can successfully be depicted by the log-normal distribution for reactors R1–R3 and for R5, whereas the population in R4 displays a slightly different characteristic. Here, small cells are present at relatively high share, and normal distribution best reflects the situation. Nevertheless, concerning the irregularity in height and disposition of the illustrated bars (Fig. 5, picture R4), this distribution appears rather questionable. In general, the comparison of cell size distribution along the 5CSTR leads to the conclusion that cell size is increasing with increasing *τ* (indicated by the increasing width of the distribution function on the right side towards higher values along the cascade). Similarly, footprint area values for maximal and average cell size by trend increase with increasing *τ* (except for the discrepancy observed for R4), and the approaching of a finite maximum value, pre-defined by biological and geometrical constraints, seems to be very likely. Regarding minimal values of cell sizes estimated in the 5CSTR, it is not possible to unambiguously conclude, due to its narrow range, if this variable has a tendency to increase with increasing *τ*.

A slightly different situation occurs for the size of PHB granules (Fig. 6). They are distributed according to a log-normal distribution (R2, R3, and R5) and according to a gamma distribution in the case of R1 and R4. Here, R4 features an exception by the striking presence of small granules, probably related to the share of small cells (see leftmost bar in Fig. 6; R4). This bar (and the irregularity in bars height through the size range) raises the questions about significance and validity of such distributions. In general, the footprint areas of PHB granules are in the range of 0.01 to 1.2 μm^2^. It can be seen that in R1 the share of small PHB granules is dominant, whereas in all other CSTRs, we witness a domination of big granules. The increasing of shape width of the distribution function towards the right side (i.e., towards higher values for PHB shape area) along the reactor cascade is also evident. This is in excellent agreement with the technological designations of the individual stages of the 5CSTR (R1, nutritionally balanced cellular growth; R2, transient reactor for metabolic switch; R3–R5, PHB synthesis under growth limitation). Analogous to cell size, the footprint area values for maximal and average PHB granule size (Fig. 7, central picture) by trend increase with increasing *τ*, but, moreover, they tend to approach a finite maximal value, indicating that the rise of PHB content in the cell volume is slowing down along the 5CSTR, probably by the action of spatial limitation. A certain discrepancy for R4 is also observed regarding average and maximal granule size. Values for minimal PHB granule size are very similar (Fig. 7, central picture), thus hampering the conclusion if they increase or not with increasing *τ*.

After the separate analysis of cell size and PHB granule size, it was a logical step to investigate the ratio of these variables along the 5CSTR. For this purpose, the ratio of related sizes was expressed as the ratio of footprint areas estimated from binary images. It can be concluded that the ratio of PHB granule to cell size is increasing until R5 (results not shown). The average value of PHB-free footprint areas by trend slightly declines with higher *τ* (Fig. 7, right picture). This trend is also visible for the minimal and maximal values of this variable. Such results had to be expected, as the free space in cells gets more and more “occupied” by the synthetized PHB during the time cells move further through the 5CSTR, especially in growth-limited reactors R3–R5. Special attention should be devoted to Fig. 8 which comparatively visualizes how the average value of whole cell’s footprint area linearly increases with increasing *τ*. Simultaneously with the progression of *τ*, the average value of PHB granule area is rising in accordance with logarithmic progression, so the remaining free space in cells is decreasing. At first, the ratio of footprint areas related to PHB and whole cells significantly increases (R1 and R2); thereafter, the increase slows down (R3 and R4), and, finally, the ratio reaches a constant value (R5).

An additional attempt was made to detect the correlation between experimentally determined volumetric PHB productivity [g/(L h)] and productivity determined by footprint areas related to PHB granules (Fig. 9). In Fig. 9 (left picture), data for R4 are excluded. During the experiment, in reactor R4, a period of low glucose concentration occurred; this period includes the time point 232 h, when samples for preparing the TEM pictures were taken. This fact has significantly influenced PHB synthesis, very likely explaining why data related to this reactor show so much deviation if compared with the others. Excluding these data drastically enhances Pearson’s correlation coefficient *R* ^2^ from 0.0071 (results not shown) to 0.8863 (Fig. 9, left picture). A similar result was obtained when the specific growth rate *μ* [g/(g h)], determined by gravimetric analysis, was compared with *μ* [μm^2^/(μm^2^ h)] estimated by footprint area analysis. In this case, *R* ^2^ values amounted to 0.0507 (results not shown) and 0.8970 when data for R4 were excluded (Fig. 9, right picture).

Results summarized in Table 1 allow comparing *μ* and *π* obtained by two different methods: by standard gravimetric procedure and by estimation of footprint area binary images. Independent on the applied method, *μ* estimated for R1 (0.17 h^−1^) was highest among all reactors, with an excellent agreement between both approaches. This result is a strong indication for the correctness of assumed linear relationship between real cell/granule sizes and related measured areas (i.e., its shape size) on binary images. This is well in accordance with results presented by Mravec et al. (2016), achieved by a similar technique. Based on these results, *μ* in continuous systems can in the future be simply and quickly estimated by digital imaging of stained cells/granules and by size estimation using appropriate software like ImageJ. For that purpose, the testing and comparison of light microscopy results with TEM technique should be performed. For other reactors than R1, values for *μ* are practically zero, independent on the applied method of estimation. This is in good relation with the central idea about the function of the individual stages of the 5CSTR system. Results for *π* obtained by the two different methods (Table 1) cannot display the same numerical values, because they differ in units. To still achieve comparable results, the footprint area of cells and PHB granules obtained from SEM pictures should be related to the volume of cells/granules first and, thereafter, should be multiplied by estimated densities of related materials. After that, *π* can be calculated as usual and compared with gravimetrically obtained data. Because of cell shape changes observed along the cascade (they were more similar to an ellipsoide than to cylinder), it was not possible to approach the cell volume by cylinder volume as shown by Mravec et al. (2016). So, in our case, it was not possible to calculate volumes; hence, *π* cannot be converted to mass units.

In addition to the cells and granule sizes, it was interesting to track the number of granules per cell along the cascade; related results are presented in Fig. S1. Under the cultivation conditions applied, the number of PHB granules per cell rarely exceeds three. The average number of granules per cell was less than two (in the range 1.59–1.81 for all reactors; see Table 1). Only in a few cases that five, six, or maximum seven PHB granules were evidenced. This is in contrast to the findings of Mravec et al. (2016) who reported that the average number of granules per cell during the PHA accumulation phase varied between 10 and 15 and neither in accordance with the results of Anderson and Dawes (1990) (10 granules on average) nor with the findings of Tian et al. (2005). Furthermore, Wahl et al. (2012) reported that PHB granules were “formed in aggregated clusters of in average 2–6 granules in most cells,” but, after 1 h of cultivation under conditions favoring PHB synthesis, the number changed to 12 granules for *R. eutropha* H16 or 1 to 4 granules for *R. eutropha* HF39, respectively. It cannot be deduced if this difference is a consequence of intrinsic strain properties (PhaM and PhaP5 regulations) or, less probably, the result of the cultivation technique. In Fig. S1 (supplementary file), it can be seen that the fraction of PHB-containing cells increases with *τ* in the system, reaching a maximum of 94.6 % in the population in R5. Interestingly, the fraction of cells with one PHB granule (among all cells harboring PHB) changes through the reactor system (maximum of 63.6 % reached in R3, then decrease to 33.7 % in R5). The fraction of cells harboring two or three PHB granules shows an opposed trend than those with one granule: they reached its maximum in R4 and R5. This can be explained rather by additional granule formation in those cells harboring one granule (under PHB synthesis favoring conditions in R4 and R5) than by assuming a merging of granules. In TEM images of R4 and R5 cultures, cells with two or three granules of different sizes are often detected. In this context, it seems that the average number of granules per cell (in the range of 1.59–1.81 granules/cell for all reactors, with slight tendency to increase with increasing *τ*) does not change too much along the cascade. It happens because of the mutual “covering” of categories along the reactor cascade, because of successive shift from one category to another (caused by rising of *τ* and by shift of cell fractions with zero, one, two, or three PHB granules towards those with higher number of granules). This means that the shift to the next category is compensated by the shift from the previous category. Using only the results presented in the present work, it is not possible to unambiguously conclude if the PHB granules (i.e., the carbonosome complex containing PhaC1 synthase, different phasins PhaP1-PhaP7, PhaM protein, and PHA depolymerase enzymes) will merge after detaching from nucleoid in the late phase of synthesis.

## Electronic supplementary material

ESM 1 (PDF 568 kb)
