# Supplementary material for: Footprint area analysis of binary imaged Cupriavidus necator cells to study PHB production at balanced, transient, and limited growth conditions in a cascade process
Source: Appl Microbiol Biotechnol. 2016 Oct 3;100(23):10065–80. doi: 10.1007/s00253-016-7844-6 (PMC5102984; doi:10.1007/s00253-016-7844-6)
Supplement: Supplementary file 1 — (PDF 568 kb) [file 253_2016_7844_MOESM1_ESM.pdf]

APPLIED MICROBIOLOGY AND BIOTECHNOLOGY

SUPPLEMENTARY MATERIAL FOR

Footprint Area Analysis of Binary Imaged *Cupriavidus necator* Cells to Study PHB Production at Balanced, Transient, and Limited Growth Conditions in a Cascade Process

DENIS VADLJA[1], MARIO NOVAK[1], MARTIN KOLLER[2,3,*], GERHART BRAUNEGG[3], PREDRAG HORVAT[1]

1) Department of Biochemical Engineering, Faculty of Food Technology and Biotechnology, University of Zagreb, Pierottijeva 6, 10000 Zagreb, Croatia
2) University of Graz; Office of Research Management and Service, c/o Institute of Chemistry, NAWI Graz, Heinrichstrasse 28/III, 8010 Graz, Austria
3) ARENA Arbeitsgemeinschaft für Ressourcenschonende & Nachhaltige Technologien, Inffeldgasse 21b, 8010 Graz, Austria

* Corresponding author: Martin Koller

University of Graz; Office of Research Management and Service, c/o Institute of Chemistry, NAWI Graz, Heinrichstrasse 28/III, 8010 Graz, Austria. Telephone: +43 316 380 5463

e-mail: martin.koller@uni-graz.at

Table S1. Measuring results of size (area) estimation for *C. necator* DSM 545 cells, PHB-free part of cells and PHB granules (population from first reactor (R1) of the 5-step cascade) using by ImageJ software rearranged SEM photos.

		UNIT	Reactor R1				
	**Picture**		**R1 (1-1)**	**R1 (1-2)**	**R1 (1-3)**	**R1 (1-4)**	**Average**
**Magnification**			20,000x	20,000x	30,000x	30,000x	
**Σ (P1) Total sum of areas related to PHB granules**		[μm]^2	2.573	3.221	1.510	1.133	
**Minimal PHB granule size (area)**		[μm]^2	0.001	0.001	0.001	0.002	0.001
**Maximal PHB granule size (area)**		[μm]^2	0.222	0.244	0.194	0.140	0.200
**Average PHB granule size (area)**		[μm]^2	0.023	0.026	0.025	0.020	0.024
**Σ (P1+P2) Total sum of whole cell sizes(areas)**		[μm]^2	54.37	60.67	22.31	22.11	
**Minimal whole cell size (area)**		[μm]^2	0.071	0.130	0.143	0.212	0.139
**Maximal whole cell size (area)**		[μm]^2	1.387	1.047	1.023	0.913	1.093
**Average of whole cell sizes (areas)**		[μm]^2	0.388	0.368	0.360	0.434	0.388
**Σ (P2) Total sum of PHB-free cell parts (*i.e.*, areas related to residual biomass)**		[μm]^2	51.80	57.45	20.80	20.98	
**Minimal PHB-free cell part size (area)**		[μm]^2	0.071	0.130	0.133	0.174	0.127
**Maximal PHB-free cell part size (area)**		[μm]^2	1.311	1.033	0.937	0.773	1.014
**Average PHB-free cell part size (area)**		[μm]^2	0.370	0.348	0.335	0.411	0.366

**Total number of analyzed cells on SEM photo, (N_P)**		140	165	62	51	
**Number of cells containing PHB granules on SEM photo, (N_G)**		69	85	37	32	
**Fraction of cells with PHB granule, (N_G/N_P)**		49.29	51.52	59.68	62.75	55.81
**(N_{1G}/N_G)***		56.52	65.88	54.05	46.88	55.83
**(N_{1G}/N_P)**		27.86	33.94	32.26	29.41	30.87
**(N_{2G}/N_G)**		28.99	27.06	32.43	34.38	30.71
**(N_{2G}/N_P)**		14.29	13.94	19.35	21.57	17.29
**(N_{3G}/N_G)**		13.04	5.88	10.81	15.63	11.34
**(N_{3G}/N_P)**		6.429	3.030	6.452	9.804	6.429
**(N_{4G}/N_G)**		0	1.176	2.703	3.125	1.751
**(N_{4G}/N_P)**		0	0.606	1.613	1.961	1.045
**(N_{5G}/N_G)**		0	0	0	0	0
**(N_{5G}/N_P)**		0	0	0	0	0
**(N_{6G}/N_G)**		1.449	0	0	0	0.362
**(N_{6G}/N_P)**		0.714	0	0	0	0.179
**(N_{7G}/N_G)**		0	0	0	0	0
**(N_{7G}/N_P)**		0	0	0	0	0
**N_{8G}/N_G)**		0	0	0	0	0
**(N_{8G}/N_P)**		0	0	0	0	0
**Number of cells in division process**		6	8	1	4	

*) N_{iG} – number of cells containing „i“ granules; N_G – number of PHB containing cells in analyzed population; N_P - total number of cells analyzed based on SEM pictures.

Table S2. Results of size (area) estimation for *C. necator* DSM 545 cells, PHB-free part of cells and PHB granules (population taken from second reactor /R2/ of the 5-step cascade) using by ImageJ software rearranged SEM photos.

		UNIT	Reactor R2			
	Picture		R2 (2-1)	R2 (2-3)	R2 (2-4)	Average
**Magnification**			20,000 x	30,000 x	65,000 x	
**Σ (P1) Total sum of areas related to PHB granules**		$[\mu m]^2$	23.51	9.951	1.377	
**Minimal PHB granule size (area)**		$[\mu m]^2$	0.005	0.014	0.510	0.005
**Maximal PHB granule size (area)**		$[\mu m]^2$	0.599	0.463	0.423	0.495
**Average PHB granule size (area)**		$[\mu m]^2$	0.096	0.111	0.115	0.107
**Σ (P1+P2) Total sum of whole cell sizes(areas)**		$[\mu m]^2$	63.21	27.33	4.196	
**Minimal whole cell size (area)**		$[\mu m]^2$	0.012	0.137	0.141	0.097
**Maximal whole cell size (area)**		$[\mu m]^2$	1.088	1.309	0.997	1.131
**Average of whole cell sizes (areas)**		$[\mu m]^2$	0.331	0.385	0.466	0.394
**Σ (P2) Total sum of PHB-free cell parts (*i.e.*, areas related to residual biomass)**		$[\mu m]^2$	39.70	17.37	2.819	
**Minimal PHB-free cell part size (area)**		$[\mu m]^2$	0.012	0.097	0.141	0.083
**Maximal PHB-free cell part size (area)**		$[\mu m]^2$	0.791	1.236	0.574	0.867
**Average PHB-free cell part size (area)**		$[\mu m]^2$	0.208	0.245	0.313	0.255
**Total number of analyzed cells on SEM photo, (N_P)**			191	71	9	
**Number of cells containing PHB granules on SEM photo, (N_G)**			161	60	6	
**Fraction of cells with PHB granule, (N_G/N_P)**			84.29	84.51	66.67	78.49
**(N_{1G}/N_G)***			65.84	65.00	33.33	54.72
**(N_{1G}/N_P)**			55.50	54.93	22.22	44.22

**(N_{2G}/N_G)**		20.50	26.67	50.00	32.39
**(N_{2G}/N_P)**		17.28	22.54	33.33	24.38
**(N_{3G}/N_G)**		10.56	3.333	0	4.631
**(N_{3G}/N_P)**		8.901	2.817	0	3.906
**(N_{4G}/N_G)**		1.863	3.333	16.67	7.288
**(N_{4G}/N_P)**		1.571	2.817	11.11	5.162
**(N_{5G}/N_G)**		1.242	1.667	0	0.970
**(N_{5G}/N_P)**		1.047	1.409	0	0.819
**(N_{6G}/N_G)**		0	0	0	0
**(N_{6G}/N_P)**		0	0	0	0
**(N_{7G}/N_G)**		0	0	0	0
**(N_{7G}/N_P)**		0	0	0	0
**N_{8G}/N_G)**		0	0	0	0
**(N_{8G}/N_P)**		0	0	0	0
**Number of cells in division process**		3	5	0	

***)** N_{iG} – number of cells containing „i“ granules; N_G – number of PHB containing cells in analyzed population; N_P - total number of analyzed cells on SEM photos.

Table S3. Results of size (area) estimation for *C. necator* DSM 545 cells, PHB-free part of cells and PHB granules (population taken from third reactor /R3/ of 5-step cascade) using by ImageJ software rearranged SEM photos.

		**UNIT**	**Reactor R3**		
	**Picture**		**R3 (3-1)**	**R3 (3-2)**	**Average**
**Magnification**			20,000 x	30,000 x	
**Σ (P1) Total sum of areas related to PHB granules**		$[\mu m]^2$	8.627	6.951	
**Minimal PHB granule size (area)**		$[\mu m]^2$	0.02	0.026	0.023
**Maximal PHB granule size (area)**		$[\mu m]^2$	0.812	0.628	0.720
**Average PHB granule size (area)**		$[\mu m]^2$	0.154	0.139	0.147
**Σ (P1+P2) Total sum of whole cell sizes(areas)**		$[\mu m]^2$	19.44	15.99	
**Minimal whole cell size (area)**		$[\mu m]^2$	0.149	0.222	0.186
**Maximal whole cell size (area)**		$[\mu m]^2$	1.462	0.846	1.154
**Average of whole cell sizes (areas)**		$[\mu m]^2$	0.432	0.470	0.451
**Σ (P2) Total sum of PHB-free cell parts (*i.e.*, areas related to residual biomass)**		$[\mu m]^2$	10.81	9.038	
**Minimal PHB-free cell part size (area)**		$[\mu m]^2$	0.062	0.105	0.084
**Maximal PHB-free cell part size (area)**		$[\mu m]^2$	0.650	0.651	0.651
**Average PHB-free cell part size (area)**		$[\mu m]^2$	0.240	0.266	0.253
**Total number of analyzed cells on SEM photo, (N_P)**			45	34	
**Number of cells containing PHB granules on SEM photo, (N_G)**			38	30	
**Fraction of cells with PHB granule, (N_G/N_P)**			84.44	88.24	86.34
**(N_{1G}/N_G)***			60.53	66.67	63.60
**(N_{1G}/N_P)**			51.11	58.82	54.99

**(N_{2G}/N_G)**		34.21	13.33	23.77
**(N_{2G}/N_P)**		28.89	11.76	20.33
**(N_{3G}/N_G)**		2.63	6.667	4.649
**(N_{3G}/N_P)**		2.222	5.882	4.052
**(N_{4G}/N_G)**		2.632	13.33	7.983
**(N_{4G}/N_P)**		2.222	11.76	6.994
**(N_{5G}/N_G)**		0	0	0
**(N_{5G}/N_P)**		0	0	0
**(N_{6G}/N_G)**		0	0	0
**(N_{6G}/N_P)**		0	0	0
**(N_{7G}/N_G)**		0	0	0
**(N_{7G}/N_P)**		0	0	0
**N_{8G}/N_G)**		0	0	0
**(N_{8G}/N_P)**		0	0	0
**Number of cells in division process**		4	2	

***)** N_{iG} – number of cells containing „i“ granules; N_G – number of PHB containing cells in analyzed population; N_P - total number of analyzed cells on SEM photos.

Table S4. Measuring results of size (area) estimation for *C. necator* DSM 545 cells, PHB-free part of cells and PHB granules (population taken from fourth reactor /R4/ of the 5-step cascade) using by ImageJ software rearranged SEM photos.

		UNIT	Reactor R4		
	Picture		R4 (4-1)	R4 (4-2)	Average
**Magnification**			20,000 x	70,000 x	
**Σ (P1) Total sum of areas related to PHB granules**		$[\mu m]^2$	7.077	2.757	
**Minimal PHB granule size (area)**		$[\mu m]^2$	0.004	0.002	0.003
**Maximal PHB granule size (area)**		$[\mu m]^2$	0.775	0.649	0.712
**Average PHB granule size (area)**		$[\mu m]^2$	0.111	0.115	0.113
**Σ (P1+P2) Total sum of whole cell sizes(areas)**		$[\mu m]^2$	15.16	4.506	
**Minimal whole cell size (area)**		$[\mu m]^2$	0.019	0.005	0.012
**Maximal whole cell size (area)**		$[\mu m]^2$	1.093	0.850	0.972
**Average of whole cell sizes (areas)**		$[\mu m]^2$	0.389	0.300	0.345
**Σ (P2) Total sum of PHB-free cell parts (*i.e.*, areas related to residual biomass)**		$[\mu m]^2$	8.085	1.749	
**Minimal PHB-free cell part size (area)**		$[\mu m]^2$	0.011	0.003	0.007
**Maximal PHB-free cell part size (area)**		$[\mu m]^2$	0.544	0.264	0.404
**Average PHB-free cell part size (area)**		$[\mu m]^2$	0.207	0.117	0.162
**Total number of analyzed cells on SEM photo, (N_P)**			39	15	
**Number of cells containing PHB granules on SEM photo, (N_G)**			34	15	
**Fraction of cells with PHB granule, (N_G/N_P)**			87.18	100	93.59
**(N_{1G}/N_G)***			58.82	53.33	56.08
**(N_{1G}/N_P)**			51.28	53.33	52.31

**(N_{2G}/N_{G})**		17.65	33.33	25.49
**(N_{2G}/N_{P})**		15.38	33.33	24.36
**(N_{3G}/N_{G})**		11.76	13.33	12.55
**(N_{3G}/N_{P})**		10.26	13.33	11.80
**(N_{4G}/N_{G})**		2.941	0	1.471
**(N_{4G}/N_{P})**		2.564	0	1.282
**(N_{5G}/N_{G})**		5.882	0	2.941
**(N_{5G}/N_{P})**		5.128	0	2.564
**(N_{6G}/N_{G})**		2.941	0	1.471
**(N_{6G}/N_{P})**		2.564	0	1.282
**(N_{7G}/N_{G})**		0	0	0
**(N_{7G}/N_{P})**		0	0	0
**$N_{8G}/N_{G})$**		0	0	0
**(N_{8G}/N_{P})**		0	0	0
**Number of cells in division process**		4	0	

***)** N_{iG} – number of cells containing „i“ granules; N_{G} – number of PHB containing cells in analyzed population; N_{P} - total number of analyzed cells on SEM photos

Table S5. Measuring results of size (area) estimation for *C. necator* DSM 545 cells, PHB-free part of cells and PHB granules (population taken from fifth reactor /R5/ of the 5-step cascade) using by ImageJ software rearranged SEM photos.

		UNIT	Reactor R5		
	**Picture**		**R5 (5-4)**	**R5 (5-6)**	**Average**
**Magnification**			30,000 x	70,000 x	
**Σ (P1) Total sum of areas related to PHB granules**		$[\mu m]^2$	12.75	4.214	
**Minimal PHB granule size (area)**		$[\mu m]^2$	0.086	0.201	0.144
**Maximal PHB granule size (area)**		$[\mu m]^2$	0.915	1.034	0.975
**Average PHB granule size (area)**		$[\mu m]^2$	0.140	0.162	0.151
**Σ (P1+P2) Total sum of whole cell sizes(areas)**		$[\mu m]^2$	21.40	6.208	
**Minimal whole cell size (area)**		$[\mu m]^2$	0.191	0.052	0.122
**Maximal whole cell size (area)**		$[\mu m]^2$	1.193	1.320	1.257
**Average of whole cell sizes (areas)**		$[\mu m]^2$	0.498	0.517	0.508
**Σ (P2) Total sum of PHB-free cell parts (*i.e.*, areas related to residual biomass)**		$[\mu m]^2$	8.652	1.994	
**Minimal PHB-free cell part size (area)**		$[\mu m]^2$	0.072	0.052	0.062
**Maximal PHB-free cell part size (area)**		$[\mu m]^2$	0.498	0.286	0.392
**Average PHB-free cell part size (area)**		$[\mu m]^2$	0.201	0.166	0.184
**Total number of analyzed cells on SEM photo, (N_P)**			43	12	
**Number of cells containing PHB granules on SEM photo, (N_G)**			42	11	
**Fraction of cells with PHB granule, (N_G/N_P)**			97.67	91.67	94.67
**(N_{1G}/N_G)***			30.95	36.36	33.66
**(N_{1G}/N_P)**			30.23	33.33	31.78

**(N_{2G}/N_G)**		42.86	36.36	39.61
**(N_{2G}/N_P)**		41.86	33.33	37.60
**(N_{3G}/N_G)**		11.90	9.091	10.50
**(N_{3G}/N_P)**		11.63	8.333	9.981
**(N_{4G}/N_G)**		9.52	9.091	9.307
**(N_{4G}/N_P)**		9.302	8.333	8.818
**(N_{5G}/N_G)**		2.381	0	1.191
**(N_{5G}/N_P)**		2.326	0	1.163
**(N_{6G}/N_G)**		2.381	0	1.191
**(N_{6G}/N_P)**		2.326	0	1.163
**(N_{7G}/N_G)**		0	9.091	4.546
**(N_{7G}/N_P)**		0	8.333	4.167
**N_{8G}/N_G)**		0	0	0
**(N_{8G}/N_P)**		0	0	0
**Number of cells in division process**		0	0	

***)** N_{iG} – number of cells containing „i“ granules; N_G – number of PHB containing cells in analyzed population; N_P - total number of analyzed cells on SEM photos.

Captions Supplementary Tables

Table S1. Measuring results of size (area) estimation for *C. necator* DSM 545 cells, PHB-free part of cells and PHB granules (population from first reactor (R1) of the 5-step cascade) using by ImageJ software rearranged SEM photos.

Table S2. Results of size (area) estimation for *C. necator* DSM 545 cells, PHB-free part of cells and PHB granules (population taken from second reactor /R2/ of the 5-step cascade) using by ImageJ software rearranged SEM photos.

Table S3. Results of size (area) estimation for *C. necator* DSM 545 cells, PHB-free part of cells and PHB granules (population taken from third reactor /R3/ of 5-step cascade) using by ImageJ software rearranged SEM photos.

Table S4. Measuring results of size (area) estimation for *C. necator* DSM 545 cells, PHB-free part of cells and PHB granules (population taken from fourth reactor /R4/ of the 5-step cascade) using by ImageJ software rearranged SEM photos.

Table S5. Measuring results of size (area) estimation for *C. necator* DSM 545 cells, PHB-free part of cells and PHB granules (population taken from fifth reactor /R5/ of the 5-step cascade) using by ImageJ software rearranged SEM photos.

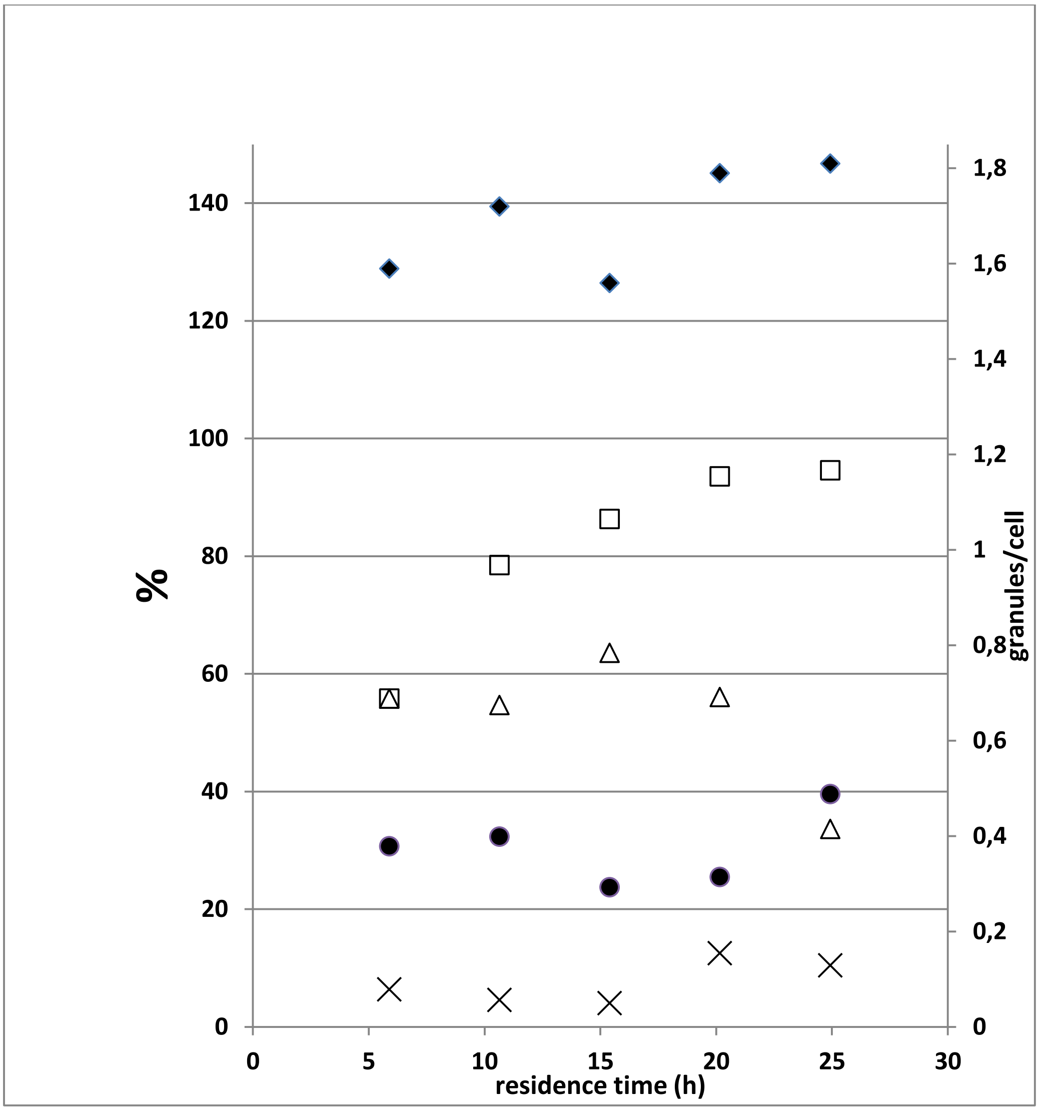

Figure S1 The influence of residence time in 5-step CSTR on average number of granules per cell (◆, right axis); and on fraction of cells that contain PHB granules (□); fractions of cells with one (Δ), two (•) and three (×) granules per cell for the population with granules, respectively (left axis).
